# Analytical Model of the Frictional Heating in a Railway Brake Disc at Single Braking with Experimental Verification

**DOI:** 10.3390/ma15196821

**Published:** 2022-10-01

**Authors:** Katarzyna Topczewska, Juraj Gerlici, Aleksander Yevtushenko, Michał Kuciej, Kateryna Kravchenko

**Affiliations:** 1 Department of Mechanics and Applied Computer Science, Faculty of Mechanical Engineering, Białystok University of Technology (BUT), 45C Wiejska Street, 15-351 Białystok, Poland; 2 Department of Transport and Handling Machines, University of Žilina, Univerzitná 8215/1, 010 26 Žilina, Slovakia

**Keywords:** railway braking system, frictional heating, temperature, analytical model, experimental investigation

## Abstract

A one-dimensional thermal problem of friction was formulated, taking into account the contact pressure increase at the beginning of the process. The obtained solution to this problem allows for the quick calculation of the transient temperature distribution in a railway brake disc during single braking application. In order to validate the developed model, the experimental tests were performed for two friction pairs consisting of the cast iron brake disc and pads comprising two composite materials. Theoretical results were compared with the data measured by thermocouples embedded in the brake disc during the full-size dynamometer tests. The maximum temperature values found based on the analytical solution are convergent with the corresponding empirical data. The consistency of the results obtained for two friction couples demonstrates the usefulness of the proposed computational model.

## 1. Introduction

One of the key development challenges in the modern railway systems is to increase the running speed of trains. This requires the improvement of the braking performance in order to maintain a short braking distance and ensure the safety of the train operation. The braking process of the railway vehicles takes place as a result of friction at the interface of the elements sliding against each other. Due to the frictional heating, brakes operate in high-temperature conditions, whichhas a significant influence on the efficiency and tribological characteristics of the braking systems. Excessive temperature results in the intensification of wear, the destabilization of the braking operation and the deterioration of the friction elements’ durability. The thermal distortion of sliding elements induces the non-uniform distribution of contact pressure and the appearance of hot spots, i.e., the local temperature rises on the friction surface and, consequently, leads to the failure of the system. Therefore, the thermal behavior of brakes during operation is the primary factor for the selection of friction materials. Experimental testing procedures are conducted on the full-size inertia dynamometers to verify the choice of friction pair materials. This kind of test allows a fairly accurate simulation of the actual railway braking system process to be achieved. In the course of the dynamometer tests, the main friction pair characteristics are determined, including measurements of temperature and the coefficient of friction and wear. The condition of the friction elements after testing is also examined, i.e., whether signs of thermal damage are observed. The temperature of the friction elements during the dynamometer test is measured using thermocouples located under the friction surface inside the elements [1]. Other than the conventional thermocouples, the so-called sliding thermocouples, which are placed directly on the friction surface, can also be found [2]. The basic disadvantage of the thermocouple methods is that the temperature values are measured only in the selected points of the system. To study the phenomena associated with the localized or uneven distribution of the temperature on the friction surface, it is necessary to use infrared thermography. This technique is successfully used in the non-destructive characterization of railway friction materials and components, as well as to detect hot spots [3]. An experimental investigation of the hot spots appearance during dynamometer tests in the railway disc brakes was carried out with a high-speed infrared camera in the study [4].

Dynamometer tests are also a stage of the process of the friction elements approval, granted by the UIC (International Union of Railways). The UIC 541-3 leaflet [5] includes general conditions for the certification of the brake pads manufactured from different materials. Alongside standard test stands, the reduced-scale dynamometers are used to study tribological phenomena related to the railway braking [6]. Researchers have noticed that the reproduction of representative full-scale conditions of thermal loading generated by friction at such a test rig is difficult; nevertheless, obtained results show that the reduced-scale tests may be representative [7]. Regardless, the performance of the experimental tests on inertia dynamometers for railway braking systems is rather complicated and expensive; therefore, the modeling of the frictional heating process is investigated to predict the thermal behavior of brakes. The basis for establishing the temperature distribution are solutions to the thermal problems of friction, i.e., the boundary-value problems of heat conduction. Mostly, the numerical methods are used, such as the finite element method (FEM), which solves even the nonlinear problems and makes it possible to include a number of interdependent quantities in the formulation of the thermal problems of friction. The advantage of the numerical methods is that they allow the actual geometry of friction elements to be introduced into the three-dimensional models.

At present, the brake discs with ventilation channels are commonly applied to improve air-cooling efficiency and ensure the stable operation of the braking systems. Rotating the disc with such geometry forces the air to flow inside the brake disc, which stimulates the heat exchange with the environment. Optimizing the disc geometry by appropriately shaping the channels significantly enhances the air-cooling effect [8]. However, the significant disadvantage of such a design is that the train movement resistance is increased. Under braking conditions, the air-pumping effect is beneficial to conventional cooling, but under running conditions the air resistance consumes the traction power of the train [9]. On the other hand, some construction elements may be helpful to reduce the air resistance effect, by preventing the air flow thought the ventilation channels of the disc during traction operation [10].

So far, various 3D FE models of frictional heating in friction elements that account for their complicated geometry have been proposed and examined [11,12,13,14,15]. A comparative numerical analysis of temperature in the solid and ventilated railway brake discs is presented in the study [14]. During the development of the calculation models, the ventilated disc has been replaced by a solid disc with the same mass and outer dimensions. Additionally, the proposed numerical models have been experimentally validated on the full-size dynamometer test stand. It has been found that considering a single braking process, simplifying the shape of the ventilated brake disc through eliminating the ventilation channels is justified [14]. This approach contributes to a significant reduction in computational time, without compromising the accuracy of the results.

Some numerical models of frictional heating have been developed, accounting for the cyclic heating and cooling of the disc rubbing surface, by modeling the brake pad as a heat source moving on the friction surface [11,14,16]. It has been found that at a fixed point of the disc, the temperature curves take the form of periodically repeating stages of rising and falling with frequency related to the disc rotations. The oscillations of the temperature during braking decline with increasing the distance from the friction surface. However, the moving heat source method takes up huge computing time for solving thermal problems during braking, which limits its wider application. To remedy this, the temperature changes in the circumferential direction can be ignored in the formulation of the problems, obtaining the axisymmetric 2D models. A comparison analysis of the three- and two-dimensional FE frictional heating models has been conducted for a railway tread brake in the study [13]. It has been proven that an axisymmetric 2D model can be almost as efficient as a 3D model to estimate the temperature distribution during repeated long-term braking mode. 

A broader review regarding the numerical modeling of frictional heating in the braking systems is presented in the article [17]. Numerical methods provide a good level of accuracy in finding the temperature; nonetheless, they allow us to obtain only approximate solutions to the problems of heat conduction. Exact solutions to such problems can be obtained by means of the analytical methods, the application of which is conditional on the adoption of appropriate simplifying assumptions. Using this approach is restricted to simplified geometric objects such as strips or semi-spaces [18]. Furthermore, nonlinear thermal problems of friction are difficult to consider using analytical methods due to the temperature-dependent friction coefficient and thermo-physical properties of materials. For this reason, these methods are more suitable for formulating the problem of heating during single braking application, in which the thermal sensitivity of friction materials can usually be neglected [19]. However, the literature contains some analytical solutions considering the thermal friction problems during repetitive, short-term braking modes [20,21,22]. The analytical techniques for the prediction of braking temperatures have significant advantages over the numerical methods: the solutions are accurate in the form of closed-form equations to calculate the temperature distributions in the friction elements during braking under specified operating parameters [18]. Compared to other techniques, analytical approaches have a higher level of flexibility and allow for a rapid and accurate computer solution of related braking problems. For the further development of analytical methods for simulating frictional heating in railway braking systems, it is necessary to verify them by performing adequate experimental tests.

The calculation scheme for determining the temperature in the railway disc brake at a repetitive short-term mode of braking has been proposed in the article [12] on the basis of the analytical methods. In addition, the corresponding results from the 3D numerical model of the ventilated brake disc have been achieved. The theoretical results have been verified based on the thermocouple measurements during the full-size dynamometer tests. It has been noted the mean temperature on the contact surfaces and the volume temperature values during intermittent braking application correlate well for these two methods. However, the results determined by the finite element method agree with experimental data slightly better than the temperatures found by the analytical method [12]. A similar analytical model for determining the mean and bulk temperature during multiple railway braking has been developed and successfully verified by means of empirical data in the study [20].

The temperature evolution of the train brake disc during high-speed braking was investigated using the infrared thermography technique, as well as the finite element modeling in the paper [23]. Moreover, the one-dimensional heat conduction model was applied to calculate the maximum temperature variations during single braking, based on the solution received in the paper [24]. It has been found that corresponding analytical results are convergent with the experimental measurements of the temperature acquired from the infrared system [23]. 

The mathematical models of the frictional heating of a brake shoe in a railway vehicle have been proposed for the continuous and emergency braking modes in the article [25]. An exact solution to the axisymmetric thermal friction problem has been obtained using the method of variables separation, in order to consider braking with constant friction power. Next, the Duhamel theorem has been introduced to find a solution for time-dependent friction power during braking operation. A similar analytical thermal analysis has been considered for a disc brake system of a railway vehicle [26]. The steady-state and transient problems of heat conduction have been formulated and solved by means of the above-mentioned methods. Another analytical model has been developed to determine the three-dimensional temperature in a brake disc of a high-speed train during the stationary braking regime in the study [16]. In order to solve the heat transfer problem, the Fourier and Hankel integral transforms have been used to perform the calculations. Obtained explicit expressions allow us to quickly compute the 3D temperature distribution in the disc brake.

This manuscript provides a new analytical model of frictional heating to establish the temperature distributions in a brake disc of the railway vehicle during single braking. The input parameters used in the calculations were adopted based on the performed experimental tests on a full-scale inertia dynamometer. The analytical calculation results show a high agreement with corresponding empirical data. 

## 2. Experimental Study

**Figure 1:**
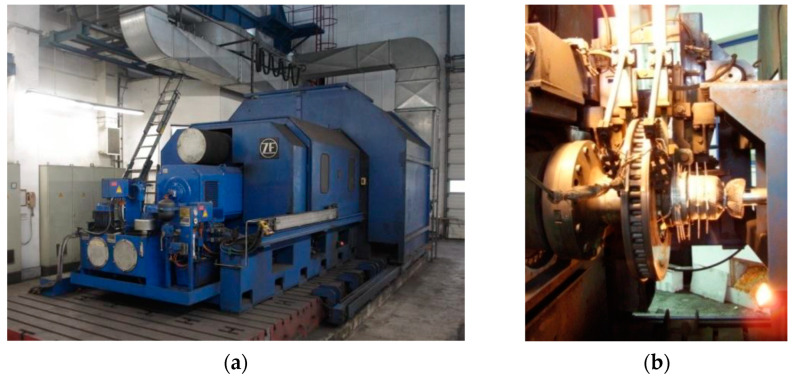
The full-scale dynamometer test stand (**a**) general view [13]; (**b**) test cabin [27].

The subject of this study is the frictional heating process in a disc brake of a railway vehicle during single braking in dry sliding conditions. The experimental tests were performed on a full-scale dynamometer test stand at the Railway Research Institute in Warsaw, Poland. The test stand is depicted in Figure 1.

**Figure 2:**
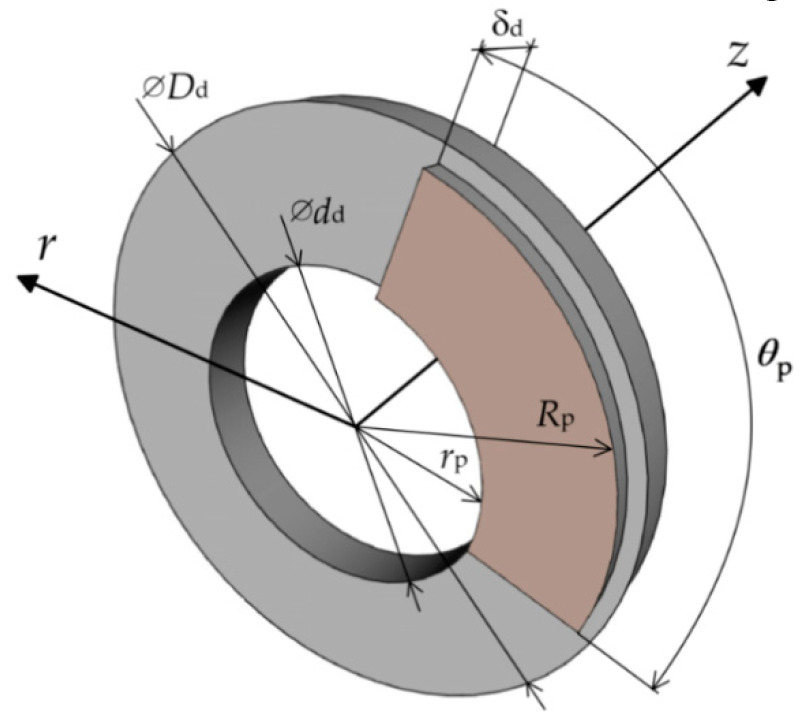
Schematic illustration of the considered friction couple.

The considered friction node consists of the brake disc and two pads positioned symmetrically on both sides of the disc. A schematic diagram of the friction couple is demonstrated in Figure 2, and the basic dimensions of the friction elements are given in Table 1.

The cast iron brake disc and two sets of brake pads were tested. The brake pads were manufactured using two different composite materials, denoted Material 874 and Material 892. The compositions of both include rubber, phenolic resins, mineral fiber, graphite, antimony sulfide and chalk. Material 892 contains less chalk that Materials 874, and it has an additional constituent—steel fiber. The weight concentration and type of the remaining ingredients of the compositions were the same for both materials. Thermal diffusivity and the specific heat and density of the pads were measured, and the thermal conductivity was calculated. The obtained thermal properties of the considered composite materials at the ambient temperature *T* = 25 °C are given in Table 2. 

During the dynamometer tests, the acquisition of temperature values reached inside the brake disc was accomplished according to the UIC 541-3 leaflet of the International Union of Railways [5]. Six thermocouples were located symmetrically on the two opposite sides in the brake disc, 1 mm under the friction surfaces to measure the temperature during braking processes. Thermocouples were installed at three different radial positions: two at the equivalent radius of braking $r_{\mathrm{eq}}$, two 40-mm outside and two 40-mm inside the equivalent radius. In the circumferential direction, the angular distance between the successive measurement locations was 120°.

The conditions simulated during the dynamometer tests correspond to the actual load and dimensions of the sliding components of the railway braking system. The experimental test procedure consists of accelerating the brake disc to the initial angular velocity $\omega_{0}$, and then braking to a standstill. The angular velocity of the disc ω was adjusted to the translational velocity V of a rail vehicle in real conditions, and it can be determined as:

(1) $$\omega (t)=\frac{V(t)}{R_{W}},\;0\leq t\leq t_{s},$$

where $R_{W}^{}$—outer radius of the vehicle wheel. At the initial moment of braking t=0 the stationary pads are pressed to the rubbing surface of the disc under the influence of the applied clamping force F. This causes the rise of pressure p on the disc pads’ contact area:

(2) $$p(t)=\frac{F(t)}{A_{a}},\;0\leq t\leq t_{s},\;$$

where $A_{a}$—nominal contact area, which in the considered system with two brake pads is equal to $A_{a}=2A_{p}$; $A_{p}$—nominal friction area of the single pad. The frictional sliding contact of elements leads to a reduction in the brake disc velocity until the stop moment $t=t_{s}$ and the brake release. The above test sequence was repeated for the second set of brake pads.

The operating parameters values were collected during the dynamometer tests. The instantaneous coefficient of friction was established by means of the following relation:

(3) $$\mu (t)=\frac{F_{t}(t)}{F(t)},\;0\leq t\leq t_{s},\;$$

where F, $F_{t}$—instantaneous contact (normal) and tangential forces, respectively.

Supposing that the friction heat is evenly distributed in the circumferential direction within the whole contact region of the disc (with cover angle 2π)*,* the calculation formula of the specific friction power q during braking on the selected radial coordinate r can be expressed as [28]:

(4) $$q(r,t)=r\mu (t)p(t)\omega (t),\;r_{p}\leq r\leq R_{p},\;0\leq t\leq t_{s},\;$$

where $r_{p}$, $R_{p}$—internal and external radiuses of the pad contact area, respectively.

## 3. Statement to the Problem

The braking processes are accompanied by the heat generation due to friction on the contact surfaces of the sliding components. Our goal is to establish the distribution of temperature in a railway brake disc. Developing the analytical calculation model of frictional heating requires adopting the assumptions that are appropriate to the conditions of a single braking process under a high sliding speed:

1. The pressure p between the disc and pads is uniformly distributed on the nominal contact area;
2. Friction materials are isotropic, and their thermo-physical properties do not vary under the influence of temperature during braking;
3. In the course of the tests, its time-averaged, constant value of mean friction coefficient f is incorporated in the calculations;
4. The influence of convective heat transfer, radiation and wear of friction surfaces on the disc temperature is neglected;
5. The whole initial kinetic energy of the vehicle is entirely converted into frictional heat on the disc-pad contact area;
6. Thermal energy generated during braking penetrates the insides of the friction couple elements along the normal direction to the friction surface, and the sum of the absorbed heat fluxes by the disc and pad is equal to the specific friction power;
7. Due to the existing symmetry of the system about the mid-plane of the disc, the analysis is conducted for the half-thickness disc in combination with one pad, supposing that the thermal processes on the other rubbing surface of the disc are the same;
8. At the initial moment of the processes, the temperature is homogeneously distributed in the elements of the friction pair.

Based on the above assumptions 1–8, the actual brake disc can be considered as a semi-infinite body z≥0, which is heated on the outer surface z=0 by the frictional heat flux. The transient temperature field T(z,t) of the element was searched from the solution of the following boundary-value problem of heat conduction [29]:

(5) $$\frac{\partial^{2}T(z,t)}{\partial z^{2}}=\frac{1}{k_{d}}\frac{\partial T(z,t)}{\partial t},\;z\geq 0,\;0\leq t\leq t_{s},$$

(6) $${K_{d}\frac{\partial T(z,t)}{\partial z}|}_{z=0}=-q_{d}(t),\;0\leq t\leq t_{s},$$

(7) $$T(z,t)\to T_{0},\;z\to \infty ,\;0\leq t\leq t_{s},$$

(8) $$T(z,0)=T_{0},\;z\geq 0,$$

where $K_{d}$, $k_{d}$—thermal conductivity and diffusivity of the disc material, $T_{0}$—initial temperature of the disc, and $q_{d}$—time-dependent intensity of heat flux absorbed by the disc. The amount of thermal energy absorbed by each element strongly depends on the thermal properties of materials. In order to establish the division of frictional heat between the elements of friction couple, the so-called heat partition ratio γ is introduced. The heat partition ratio for homogeneous materials is commonly calculated based on the known Charron’s formula [30]:

(9) $$\gamma =\frac{\sqrt{K_{d}c_{d}\rho_{d}}}{\sqrt{K_{d}c_{d}\rho_{d}}+\sqrt{K_{p}c_{p}\rho_{p}}},$$

where the material properties relating to the disc and the pads are denoted by the subscripts “d” and “p”, respectively. Then, the heat flux with intensity γq(t) is absorbed by the disc and the pad is penetrated by the heat flux with intensity (1−γ)q(t), where q(t) is the specific friction power (4). Considering the one-dimensional model of frictional heating, the power of friction generated on the contact area of the disc and pad were averaged using the coverage factor [31]:

(10) $$\eta =\frac{\theta_{p}}{2\pi },$$

where $\theta_{p}$—cover angle of the pad. Therefore, the part of thermal energy directed towards the disc in the course of the braking process can be calculated from the following dependency:

(11) $$q_{d}(t)=\eta \gamma q(t),\;0\leq t\leq t_{s}.$$

In order to obtain an exact solution to the formulated thermal problem of friction (5)–(8), the experimental data found from Equations (1)–(4), which are given in tabular form, should be described by means of the proper functions of time. Based on the measurement carried out in the course of the dynamometer tests, it was found that the pressure on the contact surface (2) rapidly grows from zero at the initial moment t=0 to the nominal value $p_{0}$ and remains until the end of braking $t=t_{s}$. The temporal profile of the contact pressure $p^{*}(t)$ was determined in the form [32]:

(12) $$p(t)=p_{0}p^{*}(t),\;p_{0}=F_{0}A_{a}^{-1},\;p^{*}(t)=\frac{t}{t_{i}}H(t_{i}-t)+H(t-t_{i}),\;0\leq t\leq t_{s},\;$$

where $p_{0}$—nominal contact pressure; $t_{i}$—time of pressure increase; H(x)—the Heaviside step function; $F_{0}$—nominal value of clamping (contact) force. 

According to the pressure (12), the velocity V of the disc can be determined from the solution to the following equation of motion:

(13) $$\frac{2W_{0}}{V_{0}^{2}}\frac{dV(t)}{dt}=-2F_{0}fp^{*}(t),\;0\leq t\leq t_{s},\;$$

with the initial condition:

(14) $$V(0)=V_{0},$$

where $W_{0}$—initial kinetic energy, $V_{0}$—initial velocity, f—mean coefficient of friction. 

The general solution to the initial problem of motion (13), (14) has the form:

(15) $$V(t)=V_{0}V^{*}(t),\;V^{*}(t)=1-\frac{1}{t_{s}^{0}}\int_{0}^{t}p^{*}(s)ds,\;t_{s}^{0}=\frac{W_{0}}{fp_{0}A_{a}V_{0}},\;0\leq t\leq t_{s}.\;$$

Substituting the profile of contact pressure (12) to the above equation (15), the profile of sliding velocity was obtained as:

(16) $$V^{*}(t)=1-\frac{1}{t_{s}^{0}}\left\{\frac{t^{2}}{2t_{i}}H(t_{i}-t)+\left[t-\frac{t_{i}}{2}\right]H(t-t_{i})\right\},\;0\leq t\leq t_{s}.$$

Based on the stop condition $V(t_{s})=0$, the time of braking $t_{s}$ was found:

(17) $$t_{s}=t_{s}^{0}+\frac{1}{2}t_{i}.$$

It should be noted that when the contact pressure rises to the nominal value immediately after the start of braking $t_{i}\to 0$, from (17) we obtained $t_{s}=t_{s}^{0}$, which means that the parameter $t_{s}^{0}$ is the time of braking in the case of braking with the constant deceleration of the vehicle.

Then, the specific friction power q(t) during braking under pressure (12) with sliding velocity (16) can be expressed as:

$$q(t)=q_{0}q^{*}(t),q_{0}=fV_{0}p_{0},$$

(18) $$q^{*}(t)=\frac{t}{t_{i}}\left(1-\frac{t^{2}}{2t_{s}^{0}t_{i}}\right)H(t_{i}-t)+\left[1-\frac{1}{t_{s}^{0}}\left(t-\frac{t_{i}}{2}\right)\right]H(t-t_{i}),\;0\leq t\leq t_{s}.$$

## 4. Solution to the Problem

The considered boundary-value problem of heat conduction (5)–(8) can be written in the following dimensionless form:

(19) $$\frac{\partial^{2}T^{*}(\zeta ,\tau )}{\partial \zeta^{2}}=\frac{\partial T^{*}(\zeta ,\tau )}{\partial \tau },\;\zeta \geq 0,\;0\leq \tau \leq \tau_{s},$$

(20) $${\frac{\partial T_{}^{*}(\zeta ,\tau )}{\partial \zeta }|}_{\zeta =0}=-q_{d}^{*}(\tau )\;,\;0\leq \tau \leq \tau_{s},$$

(21) $$T^{*}(\zeta ,\tau )\to 0,\;\zeta \to \infty ,\;0\leq \tau \leq \tau_{s},$$

(22) $$T^{*}(\zeta ,0)=0,\;\zeta \geq 0.$$

by means of the introduced dimensionless variables and parameters:

(23) $$\zeta =\frac{z}{a},\;\tau =\frac{k_{d}t}{a^{2}},\;\tau_{i}=\frac{k_{d}t_{i}}{a^{2}},\;\tau_{s}^{0}=\frac{k_{d}t_{s}^{0}}{a^{2}},\;\tau_{s}=\frac{k_{d}t_{s}}{a^{2}},\;q_{d}^{*}=\frac{q_{d}}{q_{0}},\;T^{*}=\frac{T-T_{0}}{\Theta },\;\Theta =\frac{q_{0}a}{K_{d}},\;$$

where $a=\sqrt{3k_{d}t_{s}}$ is the so-called effective depth of heat penetration in the disc [33]. 

Based on Duhamel’s theorem [34], the solution to the dimensionless thermal problem of friction (19)–(22) can be written in the form:

(24) $$T^{*}\;(\zeta ,\tau )=\int_{0}^{\tau }q_{d}^{*}(s)\frac{\partial }{\partial \tau }{\tilde{T}}^{*}(\zeta ,\tau -s)ds,\;\zeta \geq 0,\;0\leq \tau \leq \tau_{s},$$

where ${\tilde{T}}^{*}(\zeta ,\tau )$ is the known solution to the problem of heating the semi-space (19)–(22) with the assumption of the constant intensity of frictional heat flux $q_{d}^{*}=1$ [35]:

(25) $${\tilde{T}}^{*}(\zeta ,\tau )=2\sqrt{\tau }\;\mathrm{ierfc}\;\;[\zeta /(2\sqrt{\tau })],\;\zeta \geq 0,\;0\leq \tau \leq \tau_{s},\;\mathrm{ierfc}(x)=\pi^{-1/2}\exp(-x^{2})-x\;\mathrm{erfc}(x),\;\mathrm{erfc}(x)=1-\mathrm{erf}(x),$$

where  erf(x) is the Gauss error function [36]. 

The partial derivative of the function (25) was determined in the form:

(26) $$\frac{\partial }{\partial \tau }{\tilde{T}}^{*}(\zeta ,\tau -s)=\frac{\exp\{-{\left[\zeta /(2\sqrt{\tau -s})\right]}^{2}\}}{\sqrt{\pi (\tau -s)}}.\;$$

Substituting results (18), (23) and (26) to the equation (24), the following was received:

(27) $$T^{*}\;(\zeta ,\tau )=T_{i}(\zeta ,\tau )H(\tau_{i}-\tau )+[T_{i}(\zeta ,\tau )-T_{i}(\zeta ,\tau -\tau_{i})+T_{s}(\zeta ,\tau )]H(\tau -\tau_{i}),\;\;\zeta \geq 0,\;0\leq \tau \leq \tau_{s},$$

(28) $$T_{i}(\zeta ,\tau )={\left(\tau_{i}\sqrt{\pi }\right)}^{-1}\int_{0}^{\tau }\left[s-s^{3}/(2\tau_{s}^{0}\tau_{i})\right]{\sqrt{\tau -s}}^{-1}\exp\{-{\left[\zeta /(2\sqrt{\tau -s})\right]}^{2}\}ds,$$

(29) $$T_{s}(\zeta ,\tau )={\sqrt{\pi }}^{-1}\int_{\tau_{i}}^{\tau }\left[1+\tau_{i}/(2\tau_{s}^{0})-s/\tau_{s}^{0}\right]{\sqrt{\tau -s}}^{-1}\exp\{-{\left[\zeta /(2\sqrt{\tau -s})\right]}^{2}\}ds.$$

Function $T_{i}(\zeta ,\tau )$ (28) can be written as:

(30) $$T_{i}(\zeta ,\tau )=\tau_{i}^{-1}[I_{1}(\zeta ,\tau )-{\left(2\tau_{s}^{0}\tau_{i}\right)}^{-1}I_{3}(\zeta ,\tau )],\;0\leq \tau \leq \tau_{i},$$

where

(31) $$I_{n}(\zeta ,\tau )={\sqrt{\pi }}^{-1}\int_{0}^{\tau }s^{n}(\sqrt{\tau -s}{)}^{-1}\exp\{-{\left[\zeta /(2\sqrt{\tau -s})\right]}^{2}\}ds,\;n=1,\;3.$$

Using the substitution method, the integrals $I_{n}(\zeta ,\tau )$ (31) can be presented in the form:

(32) $$I_{1}(\zeta ,\tau )=2{\left(\sqrt{\pi }\right)}^{-1}[\tau L_{2}(\zeta ,\tau )-L_{4}(\zeta ,\tau )],$$

(33) $$I_{3}(\zeta ,\tau )=2{\left(\sqrt{\pi }\right)}^{-1}[L_{8}(\zeta ,\tau )-3\tau L_{6}(\zeta ,\tau )+3\tau^{2}L_{4}(\zeta ,\tau )-\tau^{3}L_{2}(\zeta ,\tau )],$$

where:

(34) $$L_{m}(\zeta ,\tau )=\int_{{\sqrt{\tau }}^{-1}}^{\infty }x^{-m}\exp[-{\left(\zeta x/2\right)}^{2}]dx,\;x={\sqrt{\tau -s}}^{-1},\;m=2,\;4,\;6,\;8.$$

Based on the solution:

(35) $$L_{2}(\zeta ,\tau )=\sqrt{\tau \pi }\;\mathrm{ierfc}\;[\zeta /(2\sqrt{\tau })],$$

and the recursive formula [37]:

(36) $$\begin{array}{l} \int u^{-l}\exp[-{\left(bu\right)}^{2}]du={\left(l-1\right)}^{-1}\{u^{1-l}\exp[-{\left(bu\right)}^{2}]-\\ \;\;\;\;\;\;\;\;\;\;\;\;\;\;\;\;\;\;\;\;\;\;\;\;\;\;\;\;\;\;\;\;\;\;\;\;\;\;\;\;\;\;\;+2b^{2}\int u^{2-l}\exp[-{\left(bu\right)}^{2}]du\},\;b>0,\;l=2,\;3\ldots , \end{array}$$

the integrals (34) for m=4, 6, 8 were found:

(37) $$L_{4}(\zeta ,\tau )=(\tau^{3/2}\sqrt{\pi }/3)\{[1-2\xi^{2}]\;\mathrm{ierfc}\;(\xi )+\xi \;\mathrm{erfc}\;(\xi )\},$$

(38) $$L_{6}(\zeta ,\tau )=(\tau^{5/2}\sqrt{\pi }/5)\;\{[1-(2/3)\xi^{2}(1-2\xi^{2})]\mathrm{ierfc}\;(\xi )+\xi [1-(2/3)\xi^{2}]\mathrm{erfc}\;(\xi )\},$$

(39) $$\begin{array}{l} L_{8}(\zeta ,\tau )=(\tau^{7/2}\sqrt{\pi }/7)\;\{[1-(2/5)\xi^{2}+(4/15)\xi^{4}(1-2\xi^{2})]\mathrm{ierfc}(\xi )+\\ \;\;\;\;\;\;\;\;\;\;\;\;\;\;\;\;\;\;\;\;\;\;\;\;\;\;\;\;\;\;\;\;\;\;\;\;\;\;\;\;\;\;\;\;\;\;\;\;\;\;\;\;\;\;+\xi [1-(2/5)\xi^{2}[1-(2/3)\xi^{2}]\mathrm{erfc}\;(\xi )\}. \end{array}$$

where:

(40) $$\xi \equiv \xi (\zeta ,\tau ),\;\xi (\zeta ,\tau )=\zeta /(2\sqrt{\tau }).$$

the variable ξ was introduced in order to simplify the notation of equations.

Taking into account the results (35), (37)–(39), the integrals (33) were found:

(41) $$I_{1}(\zeta ,\tau )=(2\tau \sqrt{\tau }/3)[2(1+\xi^{2})\mathrm{ierfc}\;(\xi )+\xi \;\mathrm{erfc}\;(\xi )],$$

(42) $$\begin{array}{l} I_{3}(\zeta ,\tau )=(2/7)\tau^{3}\sqrt{\tau }\{[16/5+(58/5)\xi^{2}+(16/3)\xi^{4}+(8/15)\xi^{6}]\mathrm{ierfc}\;(\xi )+\\ \;\;\;\;\;\;\;\;\;\;\;\;\;\;\;\;\;\;\;\;\;\;\;\;\;\;\;\;\;\;\;\;\;\;\;\;\;\;\;\;\;\;\;\;\;+(1/5)[19+12\xi^{2}+(4/3)\xi^{4}]\xi \mathrm{erfc}\;(\xi )\}. \end{array}$$

Applying results (41), (42) into the equation (29), the following was received:

(43) $$\begin{array}{l} T_{i}(\zeta ,\tau )=\frac{\tau \sqrt{\tau }}{\tau_{i}}\langle \left\{\frac{4}{3}(1+\xi^{2})-\frac{2\tau^{2}}{7\tau_{s}^{0}\tau_{i}}\left[\frac{8}{5}+\frac{29}{5}\xi^{2}+\frac{8}{3}\xi^{4}+\frac{4}{15}\xi^{6}\right]\right\}\mathrm{ierfc}\;(\xi )-\\ \;\;\;\;\;\;\;\;\;\;\;\;\;\;\;\;\;\;\;\;\;\;\;\;\;\;\;\;\;\;\;\;\;\;\;\;\;\;\;\;+\left\{\frac{2}{3}-\frac{\tau^{2}}{7\tau_{s}^{0}\tau_{i}}\left[\frac{19}{5}+\frac{12}{5}\xi^{2}+\frac{4}{15}\xi^{4}\right]\right\}\xi \;\mathrm{erfc}\;(\xi )\rangle . \end{array}$$

In order to solve the integral (30), the substitution $x={\sqrt{\tau -s}}^{-1}$ was used and the following formula was obtained:

(44) $$T_{s}(\zeta ,\tau )=[(\tau_{s}^{0}-\tau +\tau_{i}/2)L_{2}(\zeta ,\tau -\tau_{i})+L_{4}(\zeta ,\tau -\tau_{i})]/\tau_{s}^{0},\;\tau_{i}\leq \tau \leq \tau_{s}.$$

Based on the results (35), (37), the Equation (44) can be written in the form:

(45) $$\begin{array}{l} T_{s}(\zeta ,\tau )={\left(\tau_{s}^{0}\right)}^{-1}\{(2\tau_{s}^{0}-2\tau +\tau_{i})\sqrt{\tau -\tau_{i}}\mathrm{ierfc}(\xi_{i})+\\ \;\;\;\;\;\;\;\;\;\;\;\;\;\;\;\;\;\;\;\;\;\;+(2/3){\left(\tau -\tau_{i}\right)}^{3/2}[(1-2\xi_{i}{}^{2})\mathrm{ierfc}(\xi_{i})+\xi_{i}\mathrm{erfc}(\xi_{i})]\},\;\tau_{i}\leq \tau \leq \tau_{s}, \end{array}$$

where

(46) $$\xi_{i}\equiv \xi (\zeta ,\tau -\tau_{i}),\;\xi (\zeta ,\tau )=\zeta /(2\sqrt{\tau -\tau_{i}}).$$

Here, the exact solution to the formulated thermal problem of friction (5)–(8) has been found using the analytical methods. Based on the obtained results (27), (43), (45), the searched temperature field in the heated element can be presented as:

(47) $$T(z,t)=T_{0}+\eta \;\gamma \;\Theta \;T^{*}(\zeta ,\tau ),\;\zeta \geq 0,\;0\leq \tau \leq \tau_{s}.$$

## 5. Analysis

The experimental tests were performed for two (no. 1 and no. 2) braking applications with different sets of brake pads, which were made of two friction materials (denoted as 874 and 892). The dimensions of the considered friction elements and the thermal properties of their materials can be found in Table 1 and Table 2, respectively. 

Both braking processes were carried out with the same input parameters, which are demonstrated in Table 3. Therefore, the amount of thermal energy generated during the tests is the same. However, due to the differences in the properties of pad materials (Table 2) and the coefficient of friction, the dissipation of the heat during the dynamometer tests is diverse, as well as the temperature distributions. Therefore, the rest of the operating parameters for both braking processes differ. Their values, which were measured or calculated, are given in Table 4. 

Based on the developed analytical model (47), the theoretical analysis of temperature distributions during the braking processes correspond to the performed experimental tests (no. 1 and no. 2). According to the requirements from the leaflet UIC 541–3 [5], the thermocouples were located on the equivalent radius of the rubbing path *r*_eq_, and distant from that radius of 40 mm outside or inside. The analysis is focused on the maximum temperature reached during tests, so only one thermocouple measurement was taken into account, which recorded the highest temperature values. This thermocouple is located the furthest from the center of the disc, on the radial position *r*_eq_ + 40 mm, due to the highest translational sliding speed of the disc in that point. The values of initial temperature *T*_0_ used in the calculations were established based on the measurement registered at the initial moments of time. 

**Figure 3:**
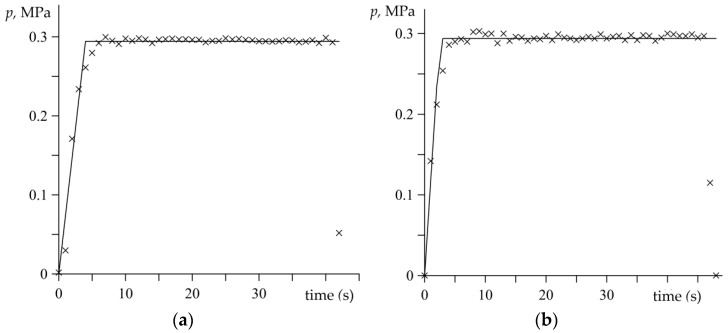
Variations of the contact pressure *p*(*t*) during tests: (**a**) no. 1; (**b**) no. 2. Comparison of the experimental data (marked with crosses) and the approximation by the function (11) (solid lines).

**Figure 4:**
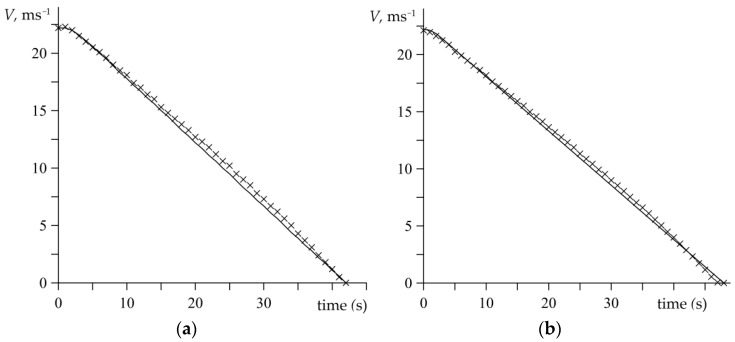
Changes of the velocity V(t) during tests: (**a**) no. 1; (**b**) no. 2. Comparison of the experimental data (marked with crosses) and the approximation by means of the function (16) (solid lines).

**Figure 5:**
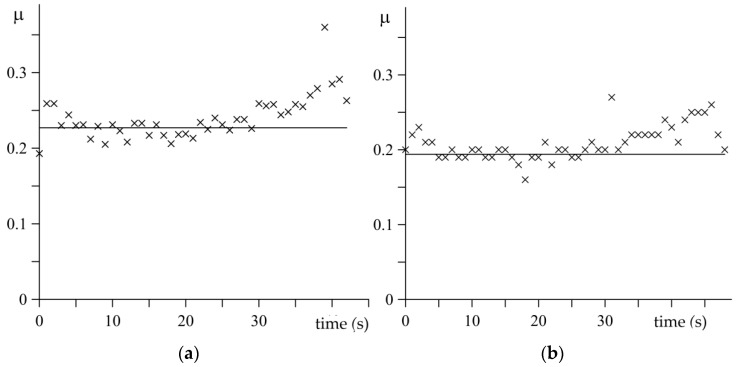
Changes of the instantaneous coefficients of friction μ(t) measured during tests (marked with crosses): (**a**) no. 1; (**b**) no. 2. Solid lines present the constant, mean values of friction coefficients.

**Figure 6:**
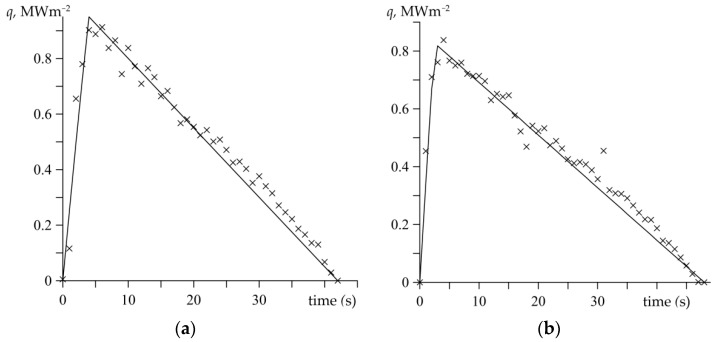
Changes of the specific friction power q(t) during processes: (**a**) no. 1; (**b**) no. 2. Comparison of the experimental data (marked with crosses) and its approximation by the function (18) (solid lines).

**Figure 7:**
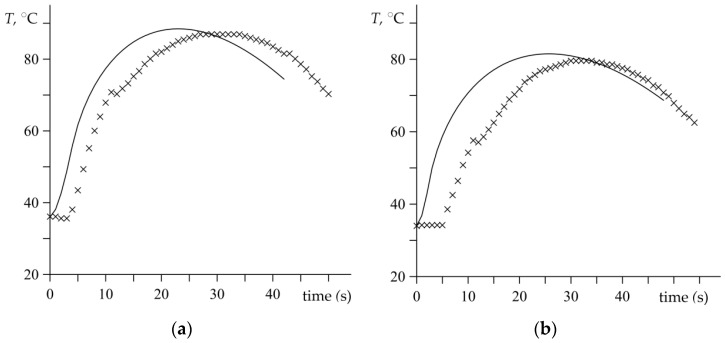
Temperature T achieved 1 mm below the friction surface during two braking applications: (**a**) no. 1; (**b**) no. 2. The experimental data (marked with crosses) and the corresponding theoretical results (solid lines).

**Figure 8:**
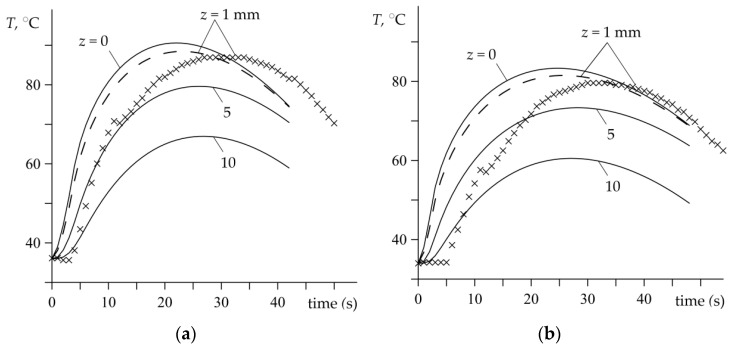
Evolutions of the temperature T on the friction surface *z* = 0 and inside the disc on a few selected depths *z* for two braking applications: (**a**) no. 1; (**b**) no. 2.

**Figure 9:**
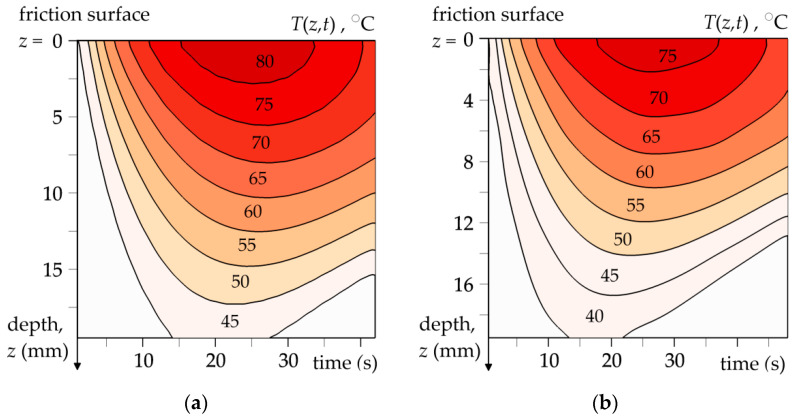
Isotherms of the temperature T(z,t) generated in the brake disc along the axial direction *z* over time of braking applications: (**a**) no. 1; (**b**) no. 2.

**Figure 10:**
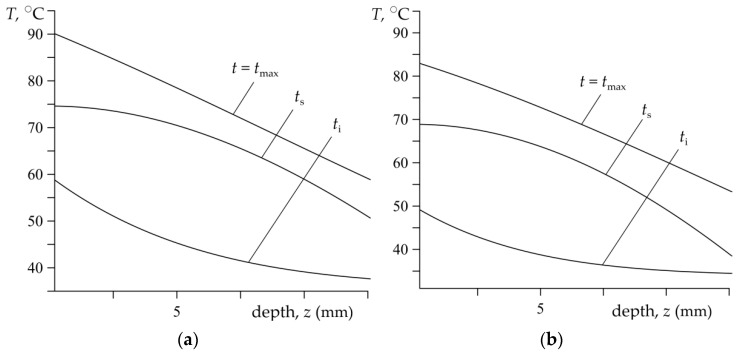
Distributions of the temperature T along the depth of the disc *z*, at the selected time moments *t* within the braking processes: (**a**) no. 1; (**b**) no. 2.

The experimental data determined in the course of the dynamometer tests, based on the relations (1)–(4), are presented in Figure 3, Figure 4, Figure 5, Figure 6, Figure 7, Figure 8, Figure 9 and Figure 10 and marked with cross-shaped points. The corresponding theoretical results that were obtained based on the developed analytical model are illustrated in Figure 3, Figure 4, Figure 5, Figure 6, Figure 7, Figure 8, Figure 9 and Figure 10 by means of the continuous lines. 

Figure 3 presents the values of pressure on the contact surface that were established based on the measurements data of the contact load from the equation (2), which is marked with crosses. Meanwhile, the solid lines demonstrate the corresponding pressure changes that were computed based on the temporal profile determined by the formula (5). At the beginning of both braking applications, the pressure on the disc–pad interface increases linearly with time from zero at the initial moment to the nominal value achieved at the moments *t*_i_. The time of pressure increase is longer in the first braking test (no. 1), which affects the course of the corresponding sliding velocity (Figure 4a). Figure 4 shows that the highest initial velocity value is maintained in the first few seconds of braking and starts to rapidly decrease after the time of pressure increase, when the pressure remains stable to the end of the processes *t*_s_ (Figure 3). Well-fitting of the velocity time profiles with the empirical data can be found in Figure 4.

The variations of the instantaneous coefficients of friction determined from relation (3) are presented in Figure 5. In the first stage of braking applications, the friction coefficient is more or less constant, close to the mean value level. After about 2/3 of the braking time, when the temperature value on the contact surface achieves a significant level, a slight increase in the value of the friction coefficient is visible in both considered braking cases (Figure 5). Regardless, during the theoretical analysis, the constant mean values of the coefficient of friction were adopted in the calculations, which are presented by the solid lines in Figure 5.

Figure 6 demonstrates the proposed functions of the specific friction power (18) to the corresponding experimental data, which are found based on formula (4). The presented specific friction power was computed for a radial position equal to *r*_eq_ + 40 mm of the considered thermocouple. The fitting of the approximation functions to the empirical data was carried out by means of the least squares method.

Evolutions of the temperature in the disc brake during braking applications (no. 1 and no. 2) are presented in Figure 7. Solid lines illustrate the theoretical results determined based on the developed analytical model (47), which are found at the point 1 mm below the friction surface of the disc. This point matches the position of the selected thermocouple. The experimental data received directly from the measurements of the thermocouple during the dynamometer tests are demonstrated in Figure 7 and marked with cross-shaped points. It can be observed that the theoretical value of temperature rapidly and immediately grows in the initial stage of the braking processes. On the other hand, the temperature measured during the dynamometer tests remain close to the initial value in the first few seconds of both braking applications. This is related to the phenomenon of the thermal inertia of the measurement thermocouples, which is described in more detail in the study [1]. 

The excellent convergence of the compared temperature values found analytically and experimentally can be seen. In the first braking test (Figure 7a), the maximum value of temperature was measured $T_{\max}=87$ °C at the time moment $t_{\max}=29\;s$, and the corresponding theoretical prediction was $T_{\max}=88.5$ °C at $t_{\max}=23\;s$. In the second process (Figure 7b), these values were $T_{\max}=79.6$ °C at $t_{\max}=32\;s$ and $T_{\max}=81.5$ °C at $t_{\max}=26\;s$, respectively, from the experimental data and theoretical calculation. The results indicate tiny differences between the values of the highest temperatures obtained experimentally and analytically, but the times of maximum temperature achievement in both braking cases are inadequate to the empirical data. It can be seen in Figure 7 that the thermocouple measurements are clearly shifted in time about 6 s, in relation to the temperature curves that are determined from the analytical results. 

Theoretically predicted evolutions of the temperature achieved on the friction surface, and at selected distances from this surface *z* = 1, 5 and 10 mm, during experimental tests is shown in Figure 8. The curve corresponding to the distance *z* = 1 mm of the thermocouple location is marked with a dashed line to indicate its relation to the presented experimental data. The maximum temperatures achieved on the contact surface are $T_{\max}=90.5$ °C and $T_{\max}=83.5$ °C, in the first and second test, respectively. Hence, the difference between the theoretical results of the maximum temperature values achieved at the friction surface and that reached at the level corresponding to the thermocouple location is equal to 2 °C. 

The spatial-temporal distributions of the temperature in the brake disc during single braking applications (no. 1 and no. 2) are illustrated by means of the isotherms in Figure 9. Figure 10 presents the distributions of temperature inside the brake disc along the axial direction for selected time moments: the time of achieving nominal contact pressure $t_{i}$, the time of maximum temperature achievement $t_{\max}$, and the stop time $t_{s}$. The results were received from the theoretical model for the input parameters corresponding with the dynamometer tests.

## 6. Conclusions

The temperature distributions in a brake disc during single braking were investigated using a theoretical analysis as well as an experimental approach. First, the simulations of two braking applications of the railway braking system were performed on the full-scale inertia dynamometer test stand for two friction materials of the brake pads. Then, the corresponding theoretical thermal analysis was carried out based on the measured data. The frictional heating model was developed based on the one-dimensional boundary-value problem of heat conduction formulated for a semi-infinite body, which was heated on its outer surface by the heat flux. An exact solution to this problem was found by means of analytical methods in the form of closed-form expressions, in order to predict the spatial-temporal distribution of the temperature. The verification of the model was performed based on a comparison of the experimental data collected from the thermocouple measurements with the appropriate computed values. The theoretical results were evaluated and found to be in agreement with the corresponding experimental data. The maximum temperature values predicted from the developed model are only 1–2 °C higher than the values obtained from the thermocouple measurements. However, due to thermal inertia, the evolutions of the theoretically established temperature were delayed in relation to the experimental data. Similar results were obtained for both tests, which were carried out for different friction pairs, confirming the high efficiency of the proposed analytical model in determining the maximum temperature during the dynamometer tests.

## Figures and Tables

**Table 1:** Basic dimensions of the friction couple elements.

Parameter, Unit	Value
external diameter of the disc *D*_d_, m	0.64
internal diameter of the disc *d*_d_, m	0.35
thickness of the disc *δ*_d_, m	0.055
cover angle of the pad *θ*_p_, rad	1.57
external radius of the pads *R*_p_, m	0.303
internal radius of the pads *r*_p_, m	0.178
equivalent radius of friction *r*_eq_, m	0.253

**Table 2:** Thermal properties of the materials at ambient temperature *T* = 25 °C.

Element	Material	Thermal Conductivity *K*, Wm^−1^K^−1^	Thermal Diffusivity *k*, m^2^s^−1^	Specific Heat *c*, J kg^−1^K^−1^	Density *ρ*, kg^−3^
disc	cast iron	51	1.437 × 10^−5^	500	7100
pad	874	2.137	1.213 × 10^−6^	986	1787
	892	2.649	1.439 × 10^−6^	899	2047

**Table 3:** Input parameters.

Parameter, Unit	Value
nominal contact force *F*_0_, kN	20
initial translational velocity *V*_0_, kmh^−1^	80
nominal pressure on the contact area *p*_0_, MPa	0.294
nominal contact area of the single pad *A*_p_, m^2^	0.034
coverage factor of the brake disc *η*	0.25
external radius of the wheel *R*_w_, m	0.435

**Table 4:** Operating parameters of the braking applications.

Parameter, Unit	No. I	No. II
heat partition ratio *γ*	0.874	0.859
time of pressure increase *t*_i_, s	4	2.5
$\mathrm{time}\;\mathrm{parameter}\;t_{s}^{0}$, s	40	46.75
time of braking *t*_s_, s	42	48
mean friction coefficient *f*	0.227	0.194
initial temperature *T*_0_, °C	36	34

## Data Availability

Data sharing is not applicable to this article.

## Nomenclature

a	Effective depth of heat penetration (m)
$A_{a}$	Nominal contact area (m^2^)
c	Specific heat of materials ($J\;{\mathrm{kg}}^{-1}K^{-1}$)
$D_{d}$	External diameter of the disc (m)
$d_{d}$	Internal diameter of the disc (m)
F	Contact force (N)
$F_{0}$	Nominal contact force (N)
$F_{t}$	Tangential force (N)
f	Mean coefficient of friction
H(⋅)	Heaviside step function
k	Thermal diffusivity of materials ($m^{2}s^{-1}$)
K	Thermal conductivity of materials ($W\;m^{-1}K^{-1}$)
p	Contact pressure (Pa)
$p_{0}$	Nominal contact pressure (Pa)
r	Radial coordinate (m)
$R_{p}$	External radius of the pad (m)
$r_{p}$	Internal radius of the pad (m)
$r_{eq}$	Equivalent radius of friction (m)
$R_{W}$	External radius of the wheel (m)
q	Intensity of heat flux ($W\;m^{-2}$)
$q_{0}$	Nominal specific power of friction ($W\;m^{-2}$)
t	Time (s)
$t_{i}$	Time of pressure increase (s)
$t_{s}^{0}$	Time of braking with constant deceleration (s)
$t_{s}$	Stop time (s)
T	Temperature (${}^{\circ }C$)
$T_{0}$	Initial temperature (${}^{\circ }C$)
$W_{0}$	Initial kinetic energy (J)
V	Sliding velocity (${\mathrm{ms}}^{-1}$)
$V_{0}$	Initial sliding velocity (${\mathrm{ms}}^{-1}$)
z	Spatial coordinate in axial direction (m )
γ	Heat partition ratio
$\delta_{d}$	Thickness of the disc (m)
$\theta_{p}$	Cover angle of the pad (rad)
$\Theta {\;}_{0}$	Scaling factor of temperature rise (${}^{\circ }C$)
η	Coverage factor of the disc rubbing surface
ρ	Density of materials ($\mathrm{kg}\;m^{-3}$)
τ	Dimensionless time
$\tau_{i}$	Dimensionless time of pressure increase
$\tau_{s}^{0}$	Dimensionless time of braking with constant deceleration
$\tau_{s}$	Dimensionless stop time
μ	Instantaneous coefficient of friction
ω	Angular speed of the brake disc (rad s^−1^)
$\omega_{0}$	Initial angular speed of the brake disc (rad s^−1^)
ζ	Dimensionless spatial coordinate in the axial direction
